# Mechanical power at a glance: a simple surrogate for volume-controlled ventilation

**DOI:** 10.1186/s40635-019-0276-8

**Published:** 2019-11-27

**Authors:** Lorenzo Giosa, Mattia Busana, Iacopo Pasticci, Matteo Bonifazi, Matteo Maria Macrì, Federica Romitti, Francesco Vassalli, Davide Chiumello, Michael Quintel, J. J. Marini, Luciano Gattinoni

**Affiliations:** 1 Departement of Anesthesiology, Emergency and Intensive Care Medicine, University of Göttingen, Göttingen, Germany; 2 SC Anestesia e Rianimazione, Ospedale San Paolo - Polo Universitario, ASST Santi Paolo e Carlo, Milan, Italy; 3 Dipartimento di Scienze Biomediche per la Salute, Centro Ricerca Coordinato di Insufficienza Respiratoria, Università degli Studi di Milano, Milan, Italy; 4 University of Minnesota and Regions Hospital, Minneapolis/St. Paul, MN USA

**Keywords:** Mechanical power, Volume-controlled ventilation, Pressure-controlled ventilation, Mathematical computation

## Abstract

**Background:**

Mechanical power is a summary variable including all the components which can possibly cause VILI (pressures, volume, flow, respiratory rate). Since the complexity of its mathematical computation is one of the major factors that delay its clinical use, we propose here a simple and easy to remember equation to estimate mechanical power under volume-controlled ventilation:

$$ \mathrm{Mechanical}\ \mathrm{Power}=\frac{\mathrm{VE}\times \left(\mathrm{Peak}\ \mathrm{Pressure}+\mathrm{PEEP}+F/6\right)}{20} $$

where the mechanical power is expressed in Joules/minute, the minute ventilation (*VE*) in liters/minute, the inspiratory flow (*F*) in liters/minute, and peak pressure and positive end-expiratory pressure (PEEP) in centimeter of water. All the components of this equation are continuously displayed by any ventilator under volume-controlled ventilation without the need for clinician intervention.

To test the accuracy of this new equation, we compared it with the reference formula of mechanical power that we proposed for volume-controlled ventilation in the past. The comparisons were made in a cohort of mechanically ventilated pigs (485 observations) and in a cohort of ICU patients (265 observations).

**Results:**

Both in pigs and in ICU patients, the correlation between our equation and the reference one was close to the identity. Indeed, the *R*^2^ ranged from 0.97 to 0.99 and the Bland-Altman showed small biases (ranging from + 0.35 to − 0.53 J/min) and proportional errors (ranging from + 0.02 to − 0.05).

**Conclusions:**

Our new equation of mechanical power for volume-controlled ventilation represents a simple and accurate alternative to the more complex ones available to date. This equation does not need any clinical intervention on the ventilator (such as an inspiratory hold) and could be easily implemented in the software of any ventilator in volume-controlled mode. This would allow the clinician to have an estimation of mechanical power at a simple glance and thus increase the clinical consciousness of this variable which is still far from being used at the bedside. Our equation carries the same limitations of all other formulas of mechanical power, the most important of which, as far as it concerns VILI prevention, are the lack of normalization and its application to the whole respiratory system (including the chest wall) and not only to the lung parenchyma.

## Introduction

**Fig. 1:**
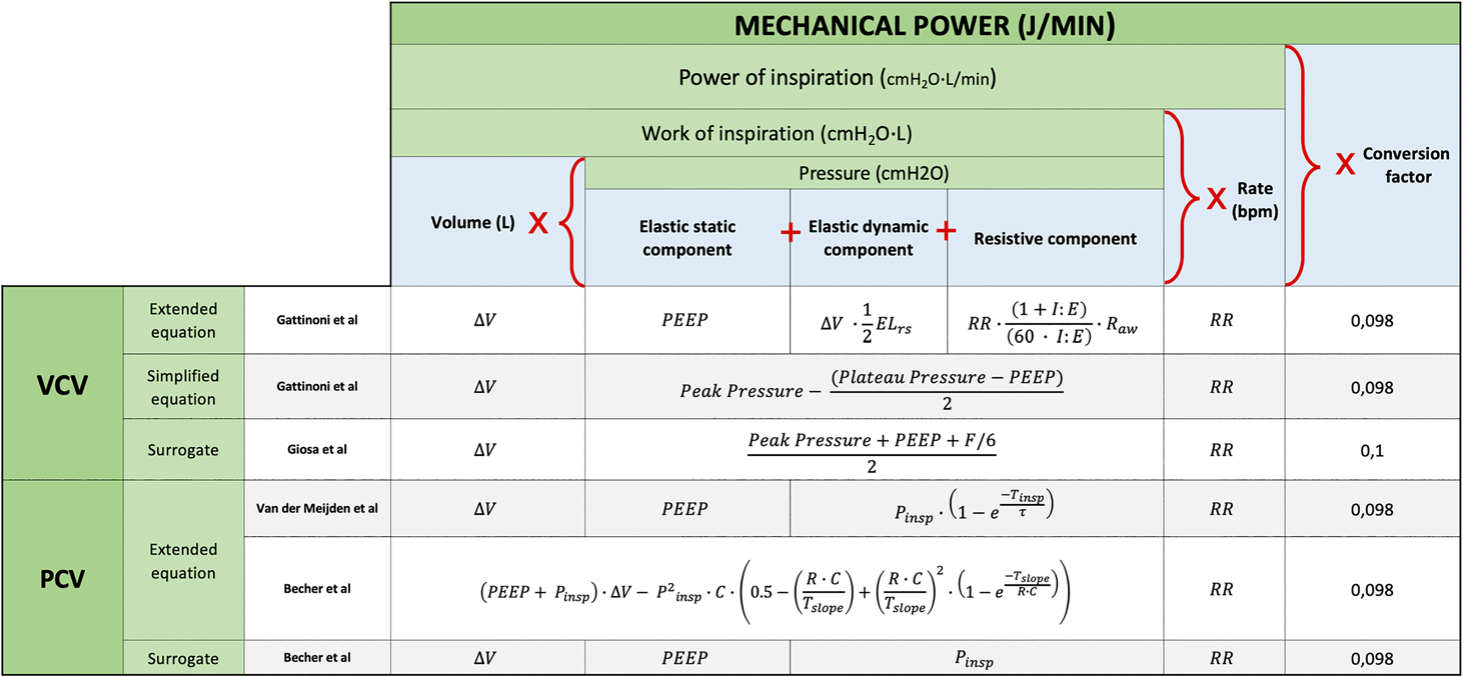
Mechanical power equations for volume-controlled and pressure-controlled ventilation. Six equations for the calculation of mechanical power are available to date. For volume-controlled ventilation, the extended equation proposed by Gattinoni et al. still represents the reference equation and the simplified equation proposed by the same group is a mathematical rearrangement of it, which means that the two formulas can be considered identical. The surrogate equation that we propose in this paper carries a small bias (underestimation), but also the advantage of being simple and easily available just by looking at the ventilator. For pressure-controlled ventilation, the two extended equations proposed by Van der Meijden et al. and by Becher et al. are both very accurate, but complex. As for our surrogate, the one proposed by Becher et al. carries a small bias (overestimation), but also the advantage of being simple and easily available just by looking at the ventilator

Mechanical power is a summary variable, which includes all the putative causes of ventilator-induced lung injury (VILI): tidal volume [1, 2], driving pressure [3] (i.e., the product of elastance and tidal volume), flow [4], respiratory rate, and end-expiratory positive pressure [5]. To better illustrate the meaning of mechanical power, we initially used the motion equation for the respiratory system multiplied by tidal volume and respiratory rate, achieving an expression of total energy delivered to the respiratory system in 1 min [6]. The advantage of using the motion equation components to illustrate the mechanical power is that the relative weight of the different variables may be clearly understood. The disadvantage is, however, the relative computational complexity. Simplified versions, however, are available [6]. Using airway pressure, these formulas apply only to relaxed patients during volume-controlled ventilation. Different versions have been proposed to compute mechanical power during passive pressure-controlled ventilation [7, 8]. A complete series of equations nowadays available are presented in Fig. 1. As illustrated, all formulas used for the computation of mechanical power are either very complex or require active manipulation of the ventilator to measure some of their constituent variables, e.g., driving pressure, airway resistance, and elastance, which all need an inspiratory hold. For volume-controlled ventilation, the extended equation proposed by Gattinoni et al. [6] represents the most precise calculation of mechanical power, but some of its variables, such as the airway and tissue resistances and the elastance of the respiratory system, are not easily measurable in the clinical setting. For pressure-controlled ventilation, two accurate equations have been proposed [7, 8], but both require the knowledge of some parameters (resistances, respiratory system compliance) which are not usually continuously displayed or quantified by the ventilator. In their paper, Becher et al. [7] also proposed a surrogate of their formula for pressure-controlled ventilation, and this equation carries the advantage of being accurate, easy to remember, and possibly continuously displayable by the ventilator. A rearrangement of the equation proposed by Gattinoni et al. [6] that simplifies the formula for mechanical power under volume-controlled ventilation is also available in the online supplement of the paper in which the extended equation is derived, but this equation still requires the application of an inspiratory hold to estimate plateau pressure. With the intent of making it easier for clinicians to estimate mechanical power under volume-controlled ventilation, we proposed an easily available and possibly continuously displayable surrogate of mechanical power (with a new simple equation) and investigated its relationship to the definitive value (calculated with the current validated equation) in two experimental cohorts of mechanically ventilated pigs and in a cohort of ICU patients.

## Methods

### Animal experiments

Data were analyzed from 77 female piglets enrolled in two different experiments [5] (unpublished data, submitted manuscript). In the first experiment, 36 piglets were mechanically ventilated for 50 h with a respiratory rate of 30 breaths per minute and a tidal volume equal to the functional residual capacity. Data from these animals were then divided into 6 groups according to the level of PEEP applied (0, 4, 7, 11, 14, 18 cmH_2_O).

In the second experiment, 42 piglets were mechanically ventilated for 48 h with different settings aiming to reach two values of mechanical power (respectively 15 and 30 J/min). Three different settings (tidal volume, respiratory rate, and PEEP) were adjusted to attain these two targeted values of mechanical power.

In these two experiments, 766 determinations of mechanical power were available, ranging from 3.96 J/min to 60.90 J/min. Since these data came from experiments whose aim was to push mechanical ventilation beyond the clinical range of mechanical power, we decided to narrow our analysis to the range of mechanical power across which 95% of ICU patients have been reported to be ventilated (11.7–31.2 J/min) [9]. This allowed us to more closely mimic clinical applicability and led the number of observations to decrease from 766 to 485. Both experiments were approved by the local authority and performed according to the European Union guidelines. The anesthesia was induced and maintained with propofol 2% (continuous infusion of 5–8 mg/kg), sufentanil (2–3 μg/kg/h), and midazolam (2 mg/kg/h). After the induction of anesthesia, piglets were intubated and mechanically ventilated in a prone position with a volume-controlled mode (constant flow). After an esophageal balloon was introduced, its correct position was adjusted and checked through the compression of the thorax during an expiratory hold. Respiratory mechanics data were collected every 6 hours until the end of the experiment or the death of the animal.

### Clinical data

Observational data from 200 ICU patients enrolled in 7 previously published studies were analyzed [10–16]. The institutional review board of each hospital approved each study, and a written consent was obtained according to the regulations applied in each Institution.

All patients were supported by volume-controlled ventilation, and their ventilator parameters were recorded first at a PEEP of 5 cmH_2_O and then at a PEEP of 15 cmH_2_O. Only patients who, for clinical reasons, had been treated first with a PEEP of 5 cmH_2_O and then of 15 cmH_2_O were selected, and no intervention was performed for the purpose of this study.

From this cohort, 339 determinations of mechanical power were available. The mechanical power of the respiratory system ranged from 5.55 J/min to 61.74 J/min. In order to compare these data with the ones coming from the animal experiments, we decided to narrow also this analysis to the range of mechanical power comprising 95% of the ICU patients reported by Serpa Neto et al. (11.7–31.2 J/min) [9]. This led the number of observations to decrease from 339 to 265.

### Calculation of mechanical power and its surrogate

The mechanical power was first determined based on the motion equation (see Fig. 1) with the simplified formula suggested by Gattinoni et al. [6] for volume-controlled ventilation:

$$ {\mathrm{Power}}_{\mathrm{rs}}= RR\times TV\times \left[\mathrm{Peak}\ \mathrm{Pressure}-\frac{\left(\mathrm{Plateau}\ \mathrm{Pressure}- PEEP\right)}{2}\ \right]\times 0.098 $$

**Fig. 2:**
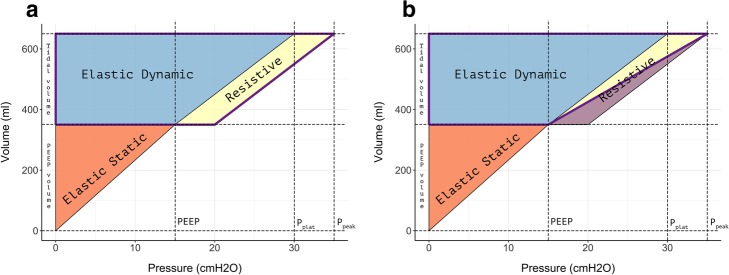
Geometrical view of mechanical power for volume-controlled ventilation. Left pane (**a**) l: the violet contoured trapezoid represents the mechanical power as calculated with the reference formula (Eq. 1). Right panel  (**b**): the violet contoured trapezoid represents the mechanical power as calculated with our unadjusted surrogate (Eq. 3). The violet-filled triangle represents the bias (Eq. 4) between our unadjusted surrogate (Eq. 3) and the reference formula (Eq. 1)

The geometrical equivalent of this equation is represented in Fig. 2, left panel, as the area of the violet trapezoid.

As a first surrogate of mechanical power, we used the following equation:

$$ {\mathrm{Power}}_{\mathrm{surr}}=\left(\frac{\mathrm{Peak}\ \mathrm{Pressure}+ PEEP}{2}\ \right)\times 0.098\times TV\times RR $$

The geometrical equivalent of this equation is shown in Fig. 2, right panel, as the area of the violet trapezoid. Equation 2 may be rearranged as follows:

$$ {\mathrm{Power}}_{\mathrm{surr}}=\frac{VE\times \left(\mathrm{Peak}\ \mathrm{Pressure}+ PEEP\right)}{20} $$

where the *VE* is the product of tidal volume and respiratory rate (*TV* × *RR*), while the value of 20 at the denominator derives from the substitution of the conversion factor between cmH_2_O∙liters and Joules (usually 0.098) with a simpler value of 0.1. Dividing this new conversion factor by 2 (which is the denominator of Eq. 2), we obtain the denominator of Eq. 3.

From Fig. 2, it is easy to see that the difference between Eqs. 1 and 2 is represented by the 50% underestimation of the resistive component (violet-filled area in Fig. 2, right panel) associated with Eq. 2. If we apply to Eq. 1 the same rearrangement on *VE* and on the conversion factor that we applied to Eq. 2, we can easily calculate the mathematical difference (bias) between Eqs. 1 and 2:

$$ \mathrm{Bias}={\mathrm{Power}}_{\mathrm{rs}}-{\mathrm{Power}}_{\mathrm{surr}}=\frac{VE\times \mathrm{Resistive}\ \mathrm{Pressure}}{20} $$

where

$$ \mathrm{Resistive}\ \mathrm{Pressure}=\mathrm{Peak}\ \mathrm{Pressure}-\mathrm{Plateau}\ \mathrm{Pressure} $$

As resistive pressure equals the product of total respiratory system resistances (*R*_*aw*_) and inspiratory flow (*F*), the bias Eq. 4 may be rewritten as follows:

$$ \mathrm{Bias}=\frac{VE\times \left({R}_{aw}\times F\right)}{20} $$

Considering that the mean value of total respiratory system resistance in mechanically ventilated patients approximates 10 cmH_2_O∙sec/liters [17–20], we introduced this value in Eq. 5. This leads to the following expression:

$$ \mathrm{Bias}=\frac{VE\times \left(10\times F\right)}{20} $$

Correcting our power surrogate adding this underestimation bias to Eq. 3, we obtain:

$$ {\mathrm{Power}}_{\mathrm{surr}.\mathrm{corr}}=\frac{VE\times \left(\mathrm{Peak}\ \mathrm{Pressure}+ PEEP+10\times F\right)}{20} $$

Since the ventilators usually express the inspiratory flow (*F*) in liters/min, we converted the units of total respiratory system resistance from cmH_2_O∙sec/liters to cmH_2_O∙min/liters. The final equation that we obtained is the following:

$$ {\mathrm{Power}}_{\mathrm{surr}.\mathrm{corr}}=\frac{VE\times \left(\mathrm{Peak}\ \mathrm{Pressure}+ PEEP+F/6\right)}{20} $$

In order to estimate the impact of higher values of respiratory system resistances in the bias between our new surrogate and the standard equation, we performed a secondary analysis on the clinical group ventilated with 15 cmH_2_O of PEEP selecting patients with respiratory system resistances higher than 15 cmH_2_O∙sec/liters. Resistances were calculated as follows:

$$ {R}_{aw}=\frac{\mathrm{Peak}\ \mathrm{Pressure}-\mathrm{Plateau}\ \mathrm{Pressure}}{F} $$

### Statistical analysis

For the data analysis, we used *R software for statistical computing*. Data are reported as mean and standard deviation or median and interquartile range, as appropriate. The relationship between the variables was investigated with a linear regression model and a Bland-Altman analysis.

## Results

### Animal experiments

**Fig. 3:**
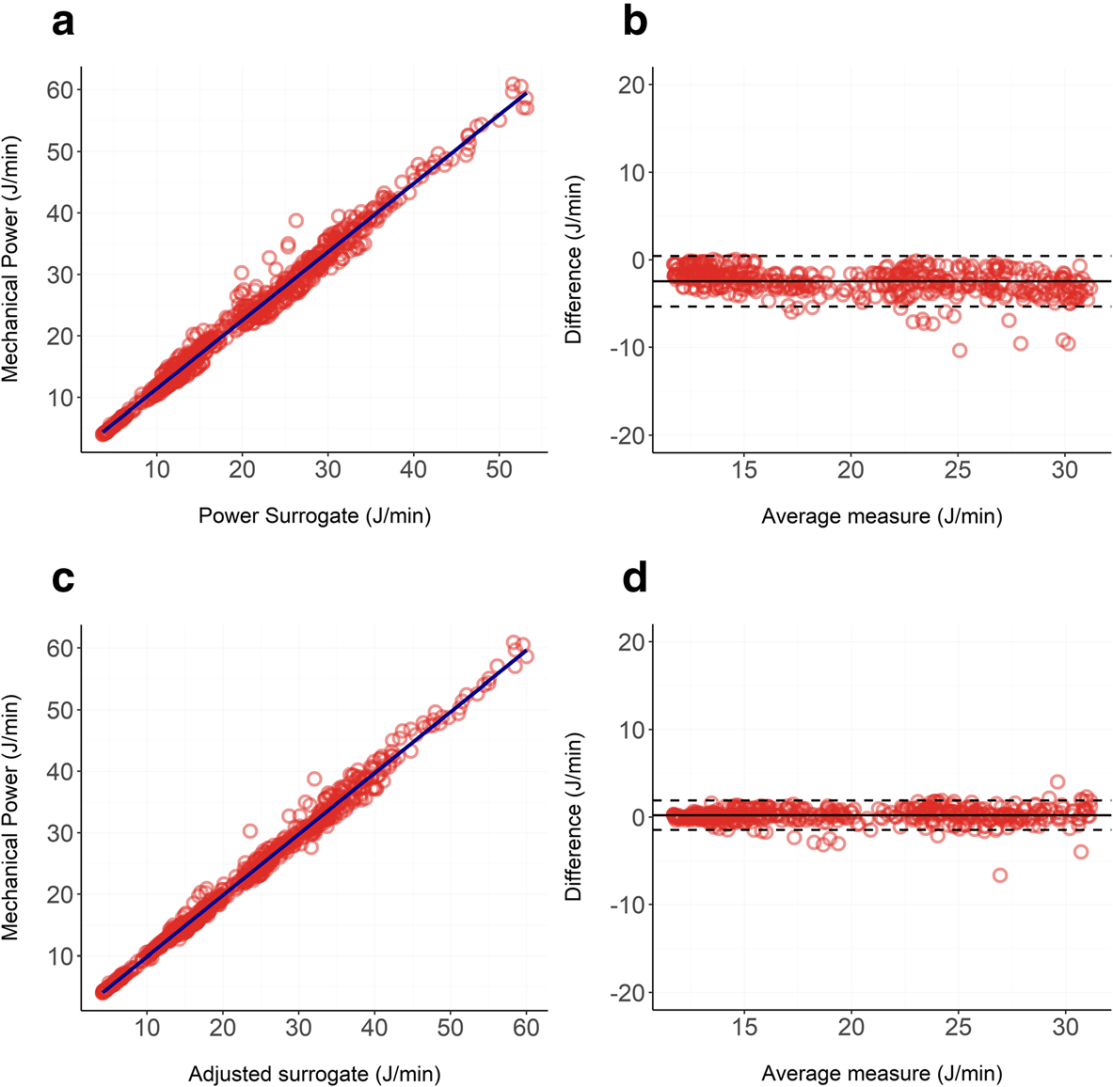
**a**, **b**: Relationship between our unadjusted surrogate of mechanical power (Eq. 3) and the reference formula (Eq. 1) in pigs: panel **a** shows the linear regression (*R*^2^ = 0.98), and panel **b** shows the Bland-Altman (bias = − 2.45 J/min; proportional error = − 0.09 J/min). **c**, **d** Relationship between our adjusted surrogate of mechanical power (Eq. 9) and the reference formula (Eq. 1) in pigs: panel **c** shows the linear regression (*R*^2^ = 0.99), and panel **d** shows the Bland-Altman (bias = 0.21 J/min; proportional error = 0.01 J/min)

Bland-Altman and linear regressions are shown in Fig. 3. The respiratory system mechanical power of our cohorts of pigs followed a non-normal distribution with a median of 22.52 J/min and an interquartile range of 17.24 J/min.

Our surrogate calculated as in Eq. 3 led to an underestimation of the mechanical power with a significant negative bias of − 2.45 J/min showing a proportional error of − 0.09 J/min for each Joules/minute of increase in mechanical power delivered. The *R*^2^ of the linear regression was 0.98 indicating an almost perfect linear relationship between the two variables. When using our adjusted surrogate (Eq. 9), the *R*^2^ was 0.99 and both the bias and the proportional error decreased to 0.21 J/min and 0.01 J/min respectively.

### Clinical data

#### Human patients ventilated with a PEEP of 5 cmH_2_0

**Fig. 4:**
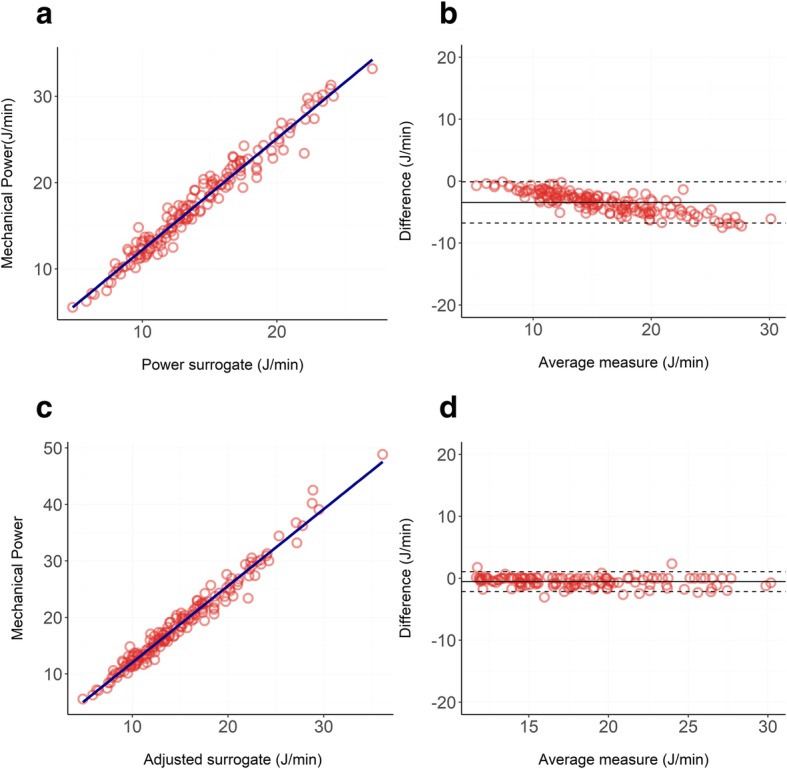
**a**, **b** Relationship between our unadjusted surrogate of mechanical power (Eq. 3) and the reference formula (Eq. 1) in ICU patients treated with 5 cmH_2_O of PEEP: panel **a** shows the linear regression (*R*^2^ = 0.96), and panel **b** shows the Bland-Altman (bias = − 3.43 J/min; proportional error = − 0.27 J/min). **c**, **d** Relationship between our adjusted surrogate of mechanical power (Eq. 9) and the reference formula (Eq. 1) in ICU patients treated with 5 cmH_2_O of PEEP: panel **c** shows the linear regression (*R*^2^ = 0.98), and panel **d** shows the Bland-Altman (bias = − 0.53 J/min; proportional error = − 0.03 J/min)

Bland-Altman and linear regressions are shown in Fig. 4. The respiratory system mechanical power followed a non-normal distribution, with a median of 17.3 J/min and an interquartile range of 9.24 J/min. Our surrogate calculated as in Eq. 3 underestimated the actual mechanical power with a significant negative bias of − 3.43 J/min, showing a proportional error of − 0.27 J/min for each Joules/minute of increase in mechanical power delivered. The *R*^2^ of the linear regression was 0.96, indicating an almost perfect linear relationship between the two variables.

When using our adjusted surrogate (Eq. 9), the *R*^2^ was 0.98 and both the underestimation bias and the proportional error decreased to − 0.534 J/min and − 0.03 J/min, respectively.

#### Human patients ventilated with a PEEP of 15 cmH_2_O

**Fig. 5:**
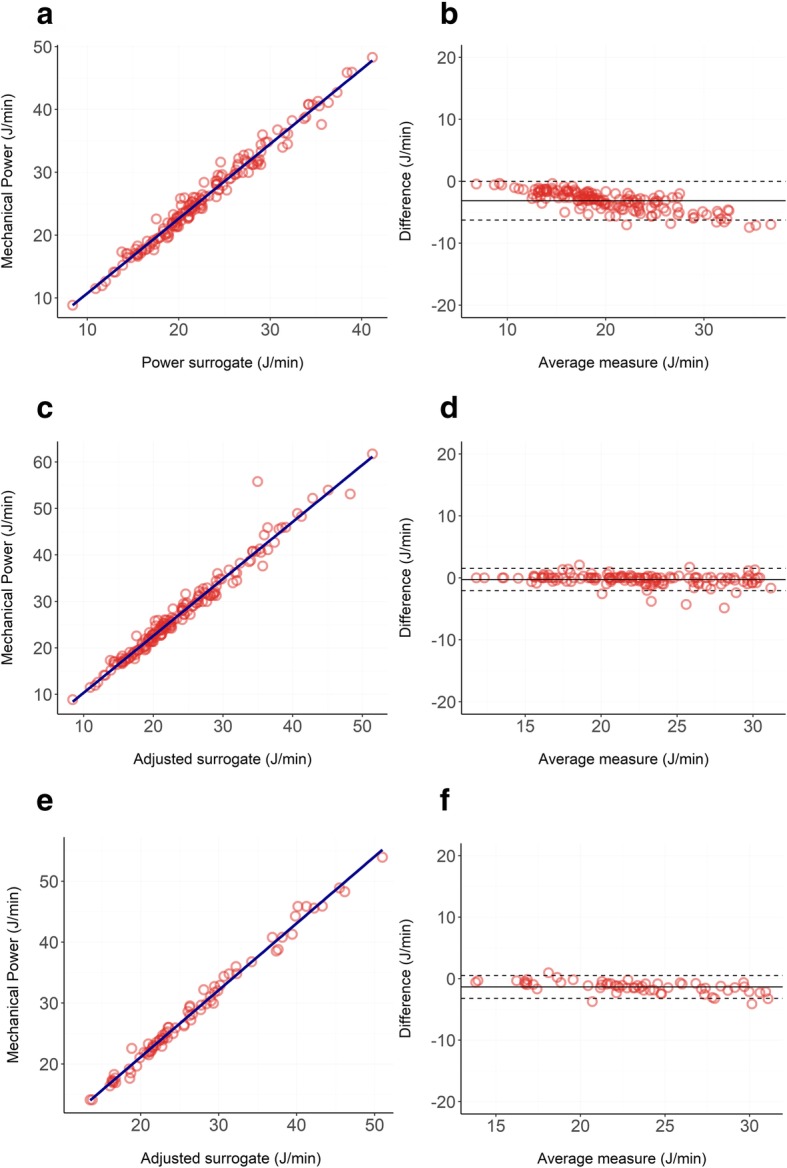
**a**, **b** Relationship between our unadjusted surrogate of mechanical power (Eq. 3) and the reference formula (Eq. 1) in ICU patients treated with 15 cmH_2_O of PEEP: panel **a** shows the linear regression (*R*^2^ = 0.97), and panel **b** shows the Bland-Altman (bias = − 3.14 J/min; proportional error = − 0.21 J/min). **c**, **d** Relationship between our adjusted surrogate of mechanical power (Eq. 9) and the reference formula (Eq. 1) in ICU patients treated with 15 cmH_2_O of PEEP: panel **c** shows the linear regression (*R*^2^ = 0.97), and panel **d** shows the Bland-Altman (bias = − 0.28 J/min; proportional error = − 0.05 J/min). **e**, **f** Relationship between our adjusted surrogate of mechanical power (Eq. 3) and the reference formula (Eq. 1) in ICU patients treated with 15 cmH_2_O of PEEP and with respiratory system resistances higher than 15 cmH_2_O∙sec/liters: panel **e** shows the linear regression (*R*^2^ = 0.98), and panel **b** shows the Bland-Altman (bias = − 1.35 J/min; proportional error = − 0.12 J/min)

Bland-Altman and linear regressions are shown in Fig. 5.

The respiratory system mechanical power followed a non-normal distribution with a median of 25.0 J/min and an interquartile range of 10.2 J/min. Our surrogate calculated as in Eq. 3 underestimated the actual mechanical power with a significant negative bias of − 3.14 J/min, showing a proportional error of − 0.21 J/min for each Joules/minute of increase in mechanical power delivered. The *R*^2^ of the linear regression was 0.97 indicating an almost perfect linear relationship between the two variables.

When using our adjusted surrogate (Eq. 9), the *R*^2^ was 0.97 and both the underestimation bias and the proportional error decreased to − 0.280 J/min and − 0.05 J/min respectively.

When narrowing the analysis to patients with respiratory system resistances higher than 15 cmH_2_O∙sec/liters we reduced the number of observations to 93. Respiratory system resistances of these patients followed a non-normal distribution, ranging from 15.02 cmH_2_O∙sec/liters to 36.6 cmH_2_O∙sec/liters, with a median value of 18.9 cmH_2_O∙sec/liters. In this population, our surrogate calculated as in Eq. 3 underestimated the actual mechanical power with a significant negative bias of − 1.35 J/min and a proportional error of − 0.12 J/min. The *R*^2^ of the linear regression was 0.98.

## Discussion

Lung injury continues to pose an important and potentially avoidable consequence of mechanical ventilation. The physiological basis of VILI is excessive strain (i.e., fractional change of volume relative to the resting volume) and its associated stress (i.e., the “counter pressure” which develops in the lung structures when a given transpulmonary pressure is applied) [21]. Estimates of stress and strain, however, are not easily available in the clinical setting [22]. Consequently, in current practice, their easily measured surrogates (tidal volume per ideal body weight and plateau pressure) are widely employed [23]. Indeed, most research on VILI has concentrated on the effects of these two variables and the standard for ventilation limits the tidal volume to 6 mL/kg [1] and the plateau pressure to 30 cmH_2_O [24]. Research on VILI, however, ranging from retrospective analyses of large databases to physiological oriented laboratory experiments, suggests that other factors such as driving pressure [3], inspiratory flow [4], and the end-expiratory pressure from which the inspiration begins [5], influence VILI development. Each of these factors, however, relates to a single breath, and although under-recognized, it is intuitively clear that the frequency and cumulative number of high-stress breathing cycles must play a substantial role in the appearance of VILI [25].

The concept of mechanical ventilating power has received remarkable attention in the scientific community. Indeed, proposals have been made to simplify its computation [6, 7], suggestions have been advanced to “normalize” it to individual patient characteristics [26], and data have been provided showing its stronger association with outcome than any of the single-breath components that comprise it: in recent experiments in which we delivered the same mechanical power by varying different only one of power’s primary components (excessive tidal volume in one group, high frequency in another group and high PEEP in the third group), we found that the lung damage was eventually similar, irrespective of the power component varied (unpublished data, manuscript submitted). If these findings are confirmed, it would follow that mechanical power or, even better, the specific mechanical power applied to the available ventilated space of the individual (i.e., somehow “normalized” for the reduced capacity of the “baby lung”) will become a key factor in assessing the VILI risk associated with mechanical ventilation. Indeed, at the moment, a value (not normalized) of 18 J/min seems to discriminate the outcome [9]. Simple methods by which it is possible to estimate mechanical power just looking at the ventilator settings and without any intervention could be of relevant clinical utility. A step forward in this field has already been made by Becher et al. for pressure-controlled ventilation [7].

In this work, we suggest that also for volume-controlled ventilation, a simple equation (Eq. 9), whose components are continuously displayed by any ventilator, can allow a reliable computation of mechanical power (throughout its clinically significant range) with an acceptably small underestimation bias. In our equation, we made two assumptions that need to be discussed: first, we approximated the conversion factor between cmH_2_O∙liters and Joules from 0.098 to 0.1. This approximation to the first decimal place makes the calculation easier and reduces the small underestimation bias which is associated with our simplified surrogate equation. Second, we assumed a fixed value of respiratory system resistance of 10 cmH_2_O∙sec/liters. This is the average value found in the literature for mechanically ventilated patients: it is clear that this assumption cannot be made for patients with a disease which primarily affects the airway resistances, such as COPD, where our new equation may underestimate the actual value of respiratory system resistances [18], and thus mechanical power. In our secondary analysis performed on patients with a median value of respiratory system resistances of 18.9 cmH_2_O∙sec/liters (almost twice the value assumed for our formula), however, we showed that the underestimation bias between our equation and the standard one remains small (− 1.35 J/min), ensuring the accuracy of our surrogate also in patients with high resistances.

The main advantage of this surrogate equation is that all of its components are continuously displayed by the ventilator under volume controlled-ventilation, obviating the need for any clinician procedure, such as imposing an inspiratory hold. As shown in Fig. 2, during volume-controlled ventilation, our mechanical power surrogate appears an adequate substitute for the standard equation so far proposed (see Fig. 1). We did not verify this simplified equation in patients treated with pressure-controlled ventilation. However, the different shapes of the pressure-volume curve under volume-controlled and pressure-controlled ventilation imply that a single equation of mechanical power would not accurately predict the energy delivered to the patient under both ventilatory modes (see Fig. 1 in the online supplement of Van der Meijden et al. [8] paper on this topic). We therefore think that the simplified equation proposed by Becher et al. [7], which is actually very similar to our unadjusted surrogate (Eq. 3), should be preferred for patients under pressure-controlled ventilation.

All the equations proposed so far to estimate mechanical power, however, do present limitations and should be considered only as first steps toward a better definition of the interplay between energy and lung. These formulas represent average inputs during inspiration and do not take into account the pathway by which the energy is applied within the single breath itself. Indeed, the intensity (i.e., Watt = Joules/sec) may be concentrated in the first part of inspiration during pressure support ventilation, whereas it could be more homogenously distributed under volume-controlled ventilation with regulated constant or sinusoidal flow. Therefore, unfortunately, our formulas do not account for the intensity of the applied mechanical power. Second, our formulas completely ignore the behavior of energy dissipation during the expiratory phase. All the elastic potential energy accumulated at the end of inspiration must be somehow released during the expiratory phase. The hysteresis area included between the inspiratory and expiratory pressure-volume loops represents the potentially dangerous energy dissipated through the airways and the lungs. It is conceivable that controlling flow during expiration, aiming to avoid abrupt early decompression and to shrink the hysteresis area, could reduce the severity of VILI, as recently suggested in experimental animals [27]. The third limitation is the dramatic lack of an easy way to “normalize” the mechanical power to the ventilable lung available. We believe that FRC measurements or the CT scan–derived estimates of aerated lung could be a reasonable way to proceed. Recently Zhang et al. [26] found that the normalization of the mechanical power to the ideal body weight appears to refine its prognostic value. However, while lung size and ideal body weight are reasonably related in patients with near-normal lungs, this relationship is completely lost in the vulnerable ARDS patients most at risk for VILI [28]. A fourth and major limitation is that all these equations, however precise, refer to the respiratory system as a whole, while the damaging mechanical power is the portion applied to individual regions within the lung parenchyma itself. While imperfect, esophageal manometry should help set a better standard for a power-focused approach to risk estimation for the lung [22]. One last limitation should be finally pointed out: referring mechanical power to the lung parenchyma and normalizing it to the FRC would only give us an estimate of the energy delivered to 1 mL of lung, but this is still far from being an accurate predictor of damage: what actually causes VILI is the microscopic local damage that develops inside the parenchyma after such energy is delivered [29]. We still have no data regarding how energy interacts with the local inhomogeneities of the lung, but this might be of high relevance in the development of VILI: when referring mechanical power to the lung parenchyma the distending pressure in the equation is not plateau pressure, but transpulmonary pressure. This pressure is often considered equal to the stress that develops inside the parenchyma when energy is applied, but the actual stress acting on the single lung unit might be up to 4 times greater than the one measured at the mouth (which is the transpulmonary pressure) [30, 31].

## Conclusions

Despite its limitations, the simple equation proposed here for volume-controlled ventilation (Eq. 9), estimated without need for clinician intervention or interruption of ventilation, may help direct more widespread attention to mechanical power in clinical practice, as well as elucidate the relationship between energy and VILI for the practitioner. Moreover, this equation is easily implementable in the software of ventilators as a continuously displayed variable under Volume-controlled ventilation.

The ideal tool to estimate the role of mechanical power on VILI would be the availability of esophageal pressure to measure the energy applied to the lung instead of the one delivered to the whole respiratory system. Unfortunately, very few centers do use this kind of measurement at the present time. Hopefully, increased understanding of the relationship between energy, lung mechanics, and VILI through a simplified approach, such as the one we propose in this paper, would provide a sufficient foundation to introduce the appropriate measurements into the normal clinical routine.

## Data Availability

The datasets used and/or analyzed during the current study are available from the corresponding author on reasonable request.

## Abbreviations

F: Inspiratory flow
FRC: Functional residual capacity
PEEP: Positive end-expiratory pressure
Raw: Resistance of the respiratory system
RR: Respiratory rate
TV: Tidal volume
VE: Minute ventilation
